# Draft genome sequence of *Pseudomona*s sp. ZS001 isolated from peanut plantation soil

**DOI:** 10.1128/mra.00658-24

**Published:** 2024-10-21

**Authors:** Chenchen Peng, Dandan Li, Haoyu Yang, Xiaoyan Wu, Xinyue Zhao, Jie Qin

**Affiliations:** 1 Tianjin Recyclable Resources Institute, All China Federation of Supply and Marketing Cooperatives, Tianjin, China; DOE Joint Genome Institute, Berkeley, California, USA

**Keywords:** *Pseudomonas*, soluble starch, biodegradation

## Abstract

*Pseudomonas* sp. ZS001 is a bacterium potentially degrading soluble starch isolated from peanut plantation soil sprayed with starch-based material in Dasi Town, Tianjin. Reporting the genome sequence of strain ZS001 can help us understand the genome composition, potential functional characteristics, and application value of *Pseudomonas* members at the genetic level.

## ANNOUNCEMENT

The genus *Pseudomonas* contains more than 200 species (1). They have been isolated from diverse sources, including soil (2), groundwater (3), sediment (4), beach sand (5), compost (6), and animal and plant tissues (7). In agricultural soil ecosystems, *Pseudomonas* spp. is a significant member of the soil microbial community, playing a regulatory role in plant growth and soil health (8). *Pseudomonas* sp. ZS001 was isolated from peanut plantation soil (stored at −80°C before incubation) collected in Tianjin Dasi (38°59′12.970″ N, 117°8′31.326″ E) in November 2023. The soil sample (2 g/L) was inoculated into LB liquid medium with 5 g/L soluble starch as inducer. After aerobic cultivation at 37°C, microbial colonies were dispersed and spread on LB solid plates. Single colonies were enriched in LB liquid medium as pure cultures. Meanwhile, the pure culture which showed good growth when transferred from LB medium to inorganic salt medium (9) with 10 g/L soluble starch as carbon source was identified as *Pseudomonas* sp. ZS001.

Pure culture of strain ZS001, stored at −80°C, was inoculated into LB liquid medium and cultured at 37°C for 12 h at 180 rpm. Briefly, 2–3 mL of the bacterial solution was utilized for genomic DNA extraction with the Bacterial DNA Extraction Kit (Majorbio, Shanghai, China) according to the manufacturer’s protocol. The 16S rRNA gene sequence was amplified using primer pair 27F/1492R (27F:5′-AGAGTTTGATCCTGGCTCAG-3′; 1492R:5′-GGTTACCTTGTTACGACTT-3′) and sequenced. The BLAST result of the ZS001 strain 16S sequence in GenBank showed that the sequences most similar to the ZS001 strain represent members of the genus *Pseudomonas* (Table 1). The DNA of strain ZS001 was used for library construction and genome sequencing. Briefly, the DNA sample was sheared into ~400-bp fragments using a Covaris M220 Focused Acoustic Shearer. The genomic DNA library was generated using the NEXTFLEX Rapid DNA-Seq Kit. The draft genome sequence was obtained on a second-generation sequencing platform (2 × 150-bp paired-end reads), Illumina NovaSeq6000, resulting in 1,122,976,430 bp of raw data (3,718,465 reads). After sequencing, low-quality reads and adapters were trimmed, bases containing non-A, G, C, and T at the 5′-end were cut, and reads with a proportion of N reaching 10% were removed with fastp software version 0.20.0 (10), followed by assembly with SOAPdenovo version 2.04 (11). Default parameters were used for all software unless otherwise specified.

**TABLE 1:** The 16S sequence identity between ZS001 strain and members of *Pseudomonas*

	Members of *Pseudomonas*	Identity (%)	Accession number
ZS001 strain	*Pseudomonas* sp. NCCP-237	99.86	AB839885.1
	*Pseudomonas piscicola* strain AC-H9	99.79	OQ421710.1
	*Pseudomonas thivervalensis* strain GHM35	99.79	OL944325.1
	*Pseudomonas lini* strain N5	99.79	KM349410.1

A total of 1,111,029,871 bp of clean data (3,661,491 reads) with a *Q*20 value of 98.47% were obtained. The draft genome sequence was assembled into 68 scaffolds with a total length of 6,006,579 bp (*N*50, 330,615 bp; G + C content, 62%). CheckM analysis version 1.2.2 (12) indicated a genome completeness of 99.9% with a contamination level of 0.21%. The genome sequence had a sequencing depth of 186.96×. In strain ZS001, 5,216 protein-coding sequences, 58 tRNAs, and 7 rRNAs were found using the NCBI Prokaryotic Genome Annotation Pipeline version 6.7 with the Best-placed reference protein set, GeneMarkS-2+. Genomic analysis of the ZS001 strain has identified putative annotated genes encoding starch degradation, desulfurization, denitrification, and antibiotic biosynthesis, indicating that this strain is a promising multifunctional environmental-adaptive bacterium.

## Data Availability

This Whole Genome Shotgun project has been deposited at DDBJ/ENA/GenBank under the accession JBCAUO000000000. The version described in this paper is version JBCAUO010000000 . The raw reads are deposited in the Sequence Read Archive (SRA) under accession number SRP515717 , BioProject accession number PRJNA1096989 , and BioSample accession number SAMN40859976.
